# Wide coverage biomedical event extraction using multiple partially overlapping corpora

**DOI:** 10.1186/1471-2105-14-175

**Published:** 2013-06-03

**Authors:** Makoto Miwa, Sampo Pyysalo, Tomoko Ohta, Sophia Ananiadou

**Affiliations:** 1 The National Centre for Text Mining and School of Computer Science, Manchester Institute of Biotechnology, University of Manchester, 131 Princess Street, Manchester, M1 7DN, UK

## Abstract

**Background:**

Biomedical events are key to understanding physiological processes and disease, and wide coverage extraction is required for comprehensive automatic analysis of statements describing biomedical systems in the literature. In turn, the training and evaluation of extraction methods requires manually annotated corpora. However, as manual annotation is time-consuming and expensive, any single event-annotated corpus can only cover a limited number of semantic types. Although combined use of several such corpora could potentially allow an extraction system to achieve broad semantic coverage, there has been little research into learning from multiple corpora with partially overlapping semantic annotation scopes.

**Results:**

We propose a method for learning from multiple corpora with partial semantic annotation overlap, and implement this method to improve our existing event extraction system, EventMine. An evaluation using seven event annotated corpora, including 65 event types in total, shows that learning from overlapping corpora can produce a single, corpus-independent, wide coverage extraction system that outperforms systems trained on single corpora and exceeds previously reported results on two established event extraction tasks from the BioNLP Shared Task 2011.

**Conclusions:**

The proposed method allows the training of a wide-coverage, state-of-the-art event extraction system from multiple corpora with partial semantic annotation overlap. The resulting single model makes broad-coverage extraction straightforward in practice by removing the need to either select a subset of compatible corpora or semantic types, or to merge results from several models trained on different individual corpora. Multi-corpus learning also allows annotation efforts to focus on covering additional semantic types, rather than aiming for exhaustive coverage in any single annotation effort, or extending the coverage of semantic types annotated in existing corpora.

## Background

**Figure 1:**
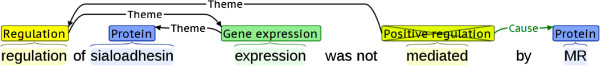
**Example event annotations.** Entities and event triggers shown with types above their corresponding text and event participants as arcs marked with roles. Each type shown in different colours. Negation visualised as “crossed-out” event.

Event extraction from biomedical literature has been the major focus of recent efforts in biomedical text mining. Events are key to understanding biological processes, and their automatic extraction facilitates the development of several domain specific applications, such as semantic search, pathway construction and database curation support [1]. Events are normally represented as *n*-ary associations of entities and other events, where participants are identified as playing specific roles (e.g., *Theme*, *Cause*) in the event. Each event is typically further associated with a *trigger* expression that denotes its occurrence in text, and may be assigned *modifiers* (attributes), marking it as being, e.g., negated (Figure 1). Established benchmark datasets and recent competitive results from multiple systems that have emerged from two shared tasks, i.e., the BioNLP Shared Task (ST) 2009 [2] and 2011 [3,4], have stimulated interest in the development of event extraction systems.

Manually created annotations are required as training material for state-of-the-art statistical systems. Manual annotation is time-consuming and expensive, and annotation efforts become increasingly demanding as more types of entities, relations and events are included in the scope of annotation. It is infeasible to deal with all potentially interesting semantic types in any single annotation effort due to cost and time restrictions and the difficulty of maintaining annotation consistency and quality while taking large numbers of semantic types into consideration. Each annotation effort thus tends to focus on a limited number of semantic types relevant to its immediate aims, which in turn results in the proliferation of corpora that overlap only partially in semantic scope, if at all [3-7].

Such partially overlapping semantic annotations represent significant challenges for statistical extraction systems. The combination of corpora whose annotation scopes are different causes difficulties in training, even in cases where semantic types shared between the corpora have been annotated using the same criteria [8].

**Figure 2:**
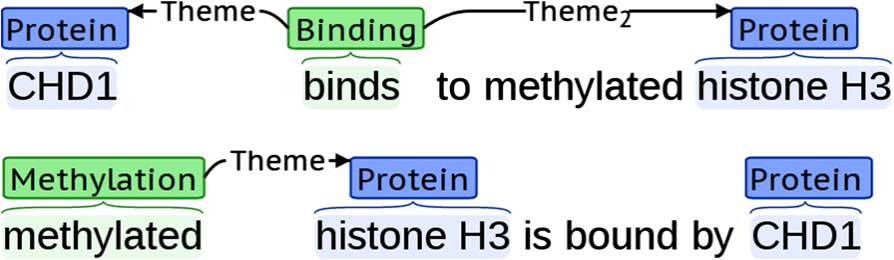
Example sentences annotated with different scopes.

As an example of the issues involved, consider Figure 2, which illustrates two example sentences arising from a hypothetical naïve combination of corpora annotated with different scopes. Given these two sentences as examples of correct annotation, a learner – whether human or machine – would have evidence that Methylation and Binding should be marked in some cases, but not in others (perhaps depending on context), and would be likely to fail to learn to mark both types of events consistently.

Due to these challenges, each system is typically trained on a single corpus that is fully annotated with a uniform set of semantic types. For multiple corpora, this results in multiple models, each of which covers only a small slice of the semantic space. In contrast to this standard setup, there has been little focus on training event extraction systems on multiple corpora. This holds also for the BioNLP STs, even though they are open challenges that explicitly allow external resources to be used in addition to the given training data.

Several previous studies have combined multiple corpora for domain-specific information extraction tasks, such as named entity (NE) recognition for NE normalisation [9] and protein-protein interaction (PPI) extraction [10,11]. However, the corpora combined in these studies contain differences not only in their annotation scopes but also in the definitions of particular semantic types. To explore the reasons for the incompatibility that exists between the semantic types in the different corpora, detailed analyses have been performed on the differences among gene and protein annotations in three common corpora [12], and on the differences among PPI annotations in five PPI corpora [13]. Despite such manual efforts to identify incompatibilities, no general automatic methods for resolving them have been introduced. Consequently, practical efforts to combine these resources rarely involve methods beyond direct corpus merging, which can show reduced performance compared to training on a single corpus.

There are also many studies on aspects of learning with domain adaptation [14], multi-task learning [15] and transfer learning [16]. As these methods make no assumptions regarding the compatibility of annotations, they can also be applied in our setting of multiple corpora with partial overlap. These types of approaches have been employed by several event extraction systems, and they have been demonstrated to improve system performance [17-21]. However, whilst assuring applicability to a wide range of problems, the lack of assumptions regarding the compatibility of annotations between different corpora also prevents them from benefiting from direct combinations of data for training. These methods also limit the scope of the resulting systems to the semantic types annotated within a single target corpus, rather than allowing the extraction of the union of types annotated in the applied corpora.

This paper focusses on the construction of a wide-coverage event extraction system by leveraging multiple corpora with partially overlapping semantic annotations as training data. In contrast to established approaches such as stacking [22] and simple domain adaptation [23], we introduce a general method that allows a single model to be trained through the merging of multiple corpora. The single model has a wide-coverage, i.e., it covers all the semantic types that appear in the multiple corpora. The method has the advantage of directly combining annotations of semantic types that are shared across different corpora, whilst also explicitly addressing the potentially negative effect of inconsistent annotation in the merged set of types that are specific only to certain corpora. Our approach is straightforward to implement and can be applied to various machine learning and information extraction tasks.

To evaluate the proposed method, we implement various approaches to corpus combination, integrate these with our existing event extraction system, EventMine [21], and perform experiments using each approach on seven biomedical event extraction corpora. Our results demonstrate that the combination of partially overlapping corpora can improve extraction performance, and that the best combination strategies can be used to train a general, wide-coverage event extraction system that outperforms systems trained on single corpora. We additionally show that using our approach, EventMine can outperform all previously proposed methods on two benchmark tasks established by the BioNLP ST 2011, the Epigenetics and Post-translational Modifications (EPI) and Infectious Diseases (ID) tasks [4]. Detailed evaluation indicates that the system can benefit from the availability of multiple corpora, not only due to a greater number of instances of shared semantic types, but also by using instances of non-shared types, which can serve as constraints in learning. The ability to learn from multiple corpora also suggests that the use of existing resources can reduce the need for the manual annotation of existing semantic types in new corpora and thus allow more efficient division of labour in annotation tasks.

## Methods

Our focus here is on the construction of a wide-coverage extraction system from multiple corpora that partially overlap in their annotation scopes, most sharing only a small number of annotated types with the other corpora in the set. In this study, we follow the BioNLP ST task setup, in which named entity annotations (e.g., *Protein*) are provided to the extraction system as part of its input, and the system aims to extract event structures that involve these named entities from text.

### Event annotated corpora

We focus on the three largest event corpora introduced in the BioNLP ST, and consider also four other corpora annotated using similar criteria. Table 1 shows the statistics of the training and development portions of the corpora.**GE** consists of texts drawn from abstracts and full texts in the *transcription factors in human blood cells* domain, annotated for nine event types involving *Protein*s [3]. The corpus was created for the BioNLP ST 2011 and is based primarily on the GENIA event corpus [24]. **ID** consists of full-text publications on the molecular mechanisms of infectious disease, annotated for the same event types as GE plus a type for high-level processes, as well as multiple entity types (e.g., *Chemical*, *Organism*). The corpus was created specifically for the BioNLP ST 2011 [4]. **EPI** consists of abstracts relating primarily to protein modifications, drawn from PubMed without other subdomain restrictions and annotated for 14 *Protein* entity modification event types and their catalysis [4]. Similarly to the ID corpus, EPI was created for the BioNLP ST 2011.**DNA methylation (DNAm)** consists of abstracts relevant to DNA methylation and demethylation events and their regulation, and is annotated for the corresponding event types. [5]. **Exhaustive PTM (EPTM)** consists of abstracts selected by relevance to a diverse set different of protein modification types, and is annotated for *Protein* entities and a comprehensive set of protein modification events. [6]. **mTOR** consists of abstracts referenced as evidence for reactions curated in the mTOR signalling pathway [25], annotated for entities and events relevant to the pathway model formalism [7]. **Multi-Level Event Extraction (MLEE)** consists of abstracts in the *blood vessel development* subdomain that have been annotated using a comprehensive set of entity and event types encompassing levels of biological organisation from molecule to organism [26].

**Table 1:** Statistics for training and development portions of applied corpora

**Corpus**	**Entities**	**Events**	**Sentences**	**Words**
GE	16,315	13,560	10,761	269,861
ID	8,501	2,779	3,412	83,063
EPI	10,094	2,453	7,827	170,809
DNAm	1,964	1,034	1,305	32,510
EPTM	4,698	1,142	3,692	82,994
mTOR	1,773	1,286	520	11,960
MLEE	3,553	4,491	1,931	37,483

**Figure 3:**
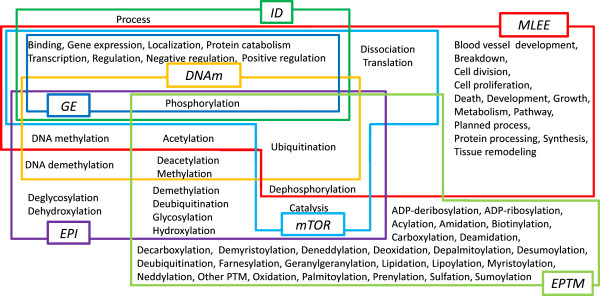
Event types annotated in event extraction corpora.

Table 2 shows the NE types annotated in these corpora. All corpora contain a *Protein* NE type, but no other NE type is shared between all corpora. Many types, such as *Regulon-operon*, *Ion* and *Cell*, are only annotated in a single corpus. Corpora other than MLEE contain also a generic *Entity* type, used primarily to mark non NE mentions (e.g., protein parts). Many event types are annotated in multiple corpora (Figure 3): for example, GE and ID corpora share all event types other than *Process*. Other corpus pairs exhibit much more modest overlap: only the *Phosphorylation* type is shared between all corpora, and EPI, DNAm and EPTM share no other type with GE and ID. In contrast, mTOR and MLEE include all of the GE event types, but add others such as *Dissociation* and *Growth*.

**Table 2:** Named entity types in applied corpora

**Corpus**	**Named entity types**
GE	Protein
ID	Protein, chemical, organism, Regulon-operon,
	two-component-system
EPI	Protein
DNAm	Protein
EPTM	Protein
mTOR	Protein, Drug, ion, simple molecule, tag
MLEE	Protein, drug or compound, cellular component, cell, tissue,
	organ, anatomical system, organism, [ …]

The partial overlap of semantic annotations bet- ween corpora is key to our approach. We build on the assumption that types sharing the same label (e.g., “*Binding*”) in two corpora denote the same semantic type and are annotated in a broadly compatible manner. For these and several other biomedical domain resources, this assumption is expected to hold due to the success of efforts such as the Gene Ontology (GO) [27] in establishing shared ontological foundations for biomedical science: all of the corpora considered in this effort draw their event types from GO and are annotated with broadly compatible criteria. This is different from corpora in many other NLP tasks, e.g., [28], which do not share ontologies, types, or annotation criteria. It should be noted that differences in scope due to entity annotation can occur even when an event type is otherwise identically annotated in two different corpora. For example, the event type *Localization* involves only *Protein* annotations in GE, but other entity types are also possible for the same event type in mTOR and MLEE. Table 3 shows the numbers of events that are transferable from one corpus to another due to the appearance of the types in both corpora.

**Table 3:** Statistics for transferable events between training and development portions of applied corpora

	**GE**	**ID**	**EPI**	**DNAm**	**EPTM**	**mTOR**	**MLEE**
GE	-	13,560	303	4,688	303	13,560	13,560
ID	1,878	-	69	524	69	1,878	1,878
EPI	130	130	-	1,668	1,988	473	1,226
DNAm	5	5	1,033	-	30	12	1,000
EPTM	85	85	315	176	-	138	155
mTOR	1,212	1,212	271	579	271	-	1,286
MLEE	2,843	2,843	49	958	38	2,852	-
SUM	6,153	17,835	2,040	8,593	2,699	18,913	19,105
RATIO	0.453	6.42	0.832	8.31	2.36	14.7	4.25

This table shows how many event instances in each row corpus can be transferred to each column corpus. SUM shows the sum of event instances in other corpora transferable to each column corpus, RATIO shows the ratio of the number of transferable event instances to the number of event instances in each column corpus. For the number of event instances in each row corpus, please refer to Table 1.

### Event extraction system: EventMine

**Figure 4:**
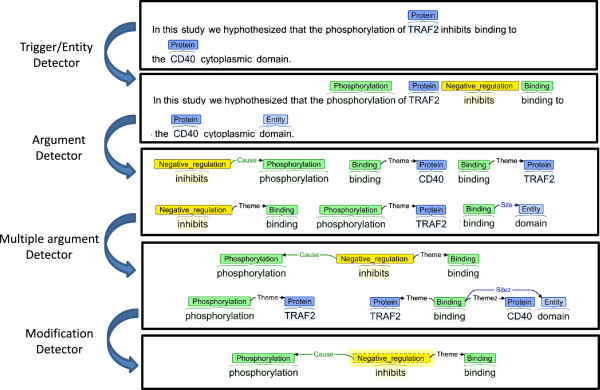
Example eventMine pipeline.

Before introducing the approach for learning from multiple corpora, we briefly introduce the baseline state-of-the-art, pipeline-based event extraction system, EventMine [21]. The system consists of four modules: trigger/entity detector, argument detector, multiple argument detector and hedge detector. Figure 4 shows an example analysis using the EventMine pipeline. Each module employs SVM classifiers with a one-vs-rest scheme, using rich features derived from a full syntactic analysis of the input text, represented using dependency-type relations.

The classifiers of each module are constructed as follows.

### The trigger/entity detector

constructs a trigger/entity word dictionary from the training data and optional external dictionaries, selects trigger/entity candidate words from all the words in texts using dictionary matching, and builds classifiers using the word candidates as instances and their types (and the negative label NONE) as labels. Note that this dictionary matching is used both in the training and prediction to reduce the computational cost, and this is separated from our filtering method which will be presented in the next section. Features for word candidates include character n-grams, context word n-grams and the shortest dependency paths between word candidates and named entities.

### The argument detector

identifies relations between trigger words detected by the trigger detector and their candidate role arguments, and builds classifiers with relations as instances, and role types and NONE as labels. Features include character n-grams in candidate participant text spans (triggers and entities), context word n-grams around the candidate participants, shortest paths between the participants, and shortest paths between the participants and other triggers or entities.

### The multiple argument detector

constructs candidate event structures by enumerating all possible combinations of the detected relations, and builds classifiers with candidate structures as instances, and event types and NONE as labels. Features are derived from the participant relations of the candidate event and other relations that include the same participants.

### The hedge detector

builds a hedge classifier with events as instances and their hedge types (*Negation*, *Speculation* and NONE in the resources considered here) as labels. Features include dependency paths containing the event trigger and relation features derived from participant relations.

Candidate construction is performed identically in training and prediction. For further details on EventMine, please see [21].

We note that despite substantial differences in implementation details, this general architecture and approach to the integration of machine learning-based classifiers is fairly common in state-of-the-art event extraction systems (e.g., [8,29]). Our approach is thus directly applicable to systems other than EventMine, and similarly it is expected that our results can be generalised to a number of other systems.

### Learning from multiple partially overlapping corpora

In conventional applications of statistical classification-based systems such as EventMine, candidate instances not annotated in the source corpus generate negative examples for training (closed-world assumption). Direct application of a system developed for training on a single corpus to multiple, partially overlapping corpora can thus lead to the creation of spurious negative instances from one corpus for cases that correspond to positive instances in terms of the scope of another corpus.

**Figure 5:**
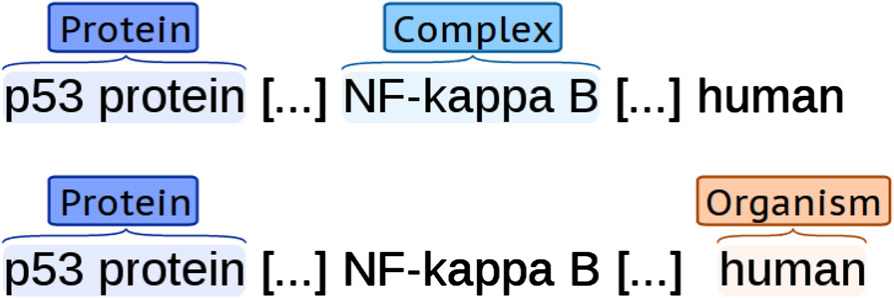
Example annotations with partially overlapping scopes.

As an example, consider the case of two corpora in which the semantic types *Protein* and *Complex* are marked in one (hereafter corpus A), whilst *Protein* and *Organism* are marked in the other (corpus B) (Figure 5). Here, *NF-kappa B* is only annotated as *Complex* in corpus A and *human* is only annotated as *Organism* in corpus B. If the corpora are directly merged and a system is trained on the merged corpus under the closed-world assumption, mentions of *human* from corpus A and and mentions of *NF-kappa B* from corpus B will be incorrectly treated as negative examples.

To avoid such negative effects from the inconsistent annotation of types that are not shared across corpora, whilst also benefiting from the annotation of shared types, it is necessary to generate from each corpus only those training instances that are relevant to types annotated in that corpus. In the setting considered here, this is straightforward – in effect automatic – for positive instances, since positive instances are always explicitly annotated. By contrast, the generation of negative examples must be restricted in a way that differentiates between valid and spurious negatives.

If there were a method to reliably determine whether a candidate instance in one corpus would have been annotated as positive under the criteria of another corpus, we could restrict the generation of negative instances in precisely the correct cases. Although there is no such general, precise, automatic method, we can automatically construct reasonably reliable filtering heuristics in the following way. For text span classification tasks (such as entity/trigger detection in event extraction), we can limit the generation of candidate negative instances in each corpus of the merged data set to only those cases in which the surface expression (or, e.g., its base form) matches at least one positive instance of an annotated type in any corpus that shares the type. For example, it is reasonable to assume that *human* never appears among the positive instances in the corpus A of the above example, and so none of the instances of the word *human* that appear in this corpus will be treated as negative instances. Analogously, for relation/event extraction, we can restrict generation to those negatives where the combination of the semantic types of the participants appears labelled as a positive instance of a type in scope of the corpus. For example, corpus A would not contain any *Protein*-*Organism* relations, and so no negative instances of this relation type would be generated for the corpus A. In cases where there are no semantic types for the participants, their surface expressions can be used instead, although this restriction may not work well when the surface expressions are diverse.

When applying a filtering approach of this type, adding new, partially overlapping corpora to the set used for training has the benefit of increasing both the positive examples of overlapping semantic types, as well as increasing the set of negative instances for these types.

We present an algorithmic description of the filtering approach in training below. 

1. Extract a set of positive (annotated) instances *P*_*j*_ from all the corpora for each type *T*_*j*_.

2. Construct a filter *F*_*j*_ (e.g., a list of surface expressions for text span classification) for each type *T*_*j*_ using the set of positive instances *P*_*j*_.

3. Construct a filter *F*_*i*_ for each corpus *C*_*i*_ from the filters for all the types annotated in *C*_*i*_

4. Extract a set of negative instances *N*_*i*_ for each corpus *C*_*i*_

(a) Extract negative instances *Ni*′ for each corpus *C*_*i*_

(b) Select *N*_*i*_ by filtering out (likely) spurious negative instances from *Ni*′ using the filter *F*_*i*_ (e.g., filter out instances if the surface expressions are not in the list generated above).

5. Train a binary classifier for each type *T*_*j*_

(a) Merge *N*_*i*_ for all *T*_*j*_-annotated corpora and all remaining positive instances for other types, i.e., *P*_*m*_ for all types *T*_*m*_ (*m*≠*j*), as negative instances *N*_*j*_

(b) Train a binary classifier on the positive instances *P*_*j*_ and the negative instances *N*_*j*_

6. Train a binary classifier for a negative type 

(a) Merge *N*_*i*_ for all corpora as positive instances *P*_*neg*_

(b) Merge *P*_*j*_ for all types as negative instances *N*_*neg*_

(c) Train a binary classifier on the positive instances *P*_*neg*_ and the negative instances *N*_*neg*_

Training must be performed in a one-vs-rest setting separately for each type (instead of a multi-class setting) as the creation of negative instances varies by type. Prediction is then performed without any filtering so that the model can extract all the semantic types in all the target corpora.

### Event extraction from multiple partially overlapping corpora

**Figure 6:**
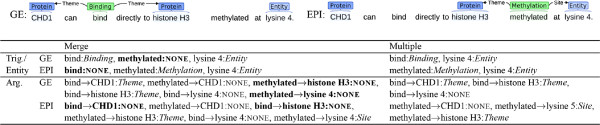
**Restriction of negative instance generation.** Different events annotated in the GE and EPI corpora are shown, along with the differences in training instances that would result in a simple merge (Merge) and our newly proposed method (Multiple) for trigger/entity and argument detectors, when all the triggers and entities are detected by the trigger/entity detectors. NONE shows negative instances, and spuriously created examples are shown in bold.

We implement the approach to filtering negative instances introduced above in the context of event extraction by modifying existing EventMine modules. Figure 6 shows an illustrative example contrasting the proposed filtering approach to a naïve corpus merge for a case that would be annotated differently for GE and EPI. The figure shows the instances that would be identified by the trigger/entity and argument detection classifiers according to the two different task descriptions (we omit other modules for brevity). Note that the sentences in the figure are prepared for illustrative purposes only; the applied corpora do not have multiple sets of annotations for any single sentence. For the trigger/entity detector, we first create for each type a merged dictionary of all the expressions (base forms) annotated with the type in any of the corpora, and then apply this to filter the candidates generated from each corpus to avoid erroneous negative instances. In Figure 6, *bind* is not generated as a negative instance (NONE label) since *bind* does not appear amongst the positive instances for EPI. Similarly, *methylation* is not amongst the negative instances. For the argument and multiple argument detectors, we determine the set of annotated semantic type combinations (e.g., *Binding*-*Protein*, *Regulation*-*Protein*-*Phosphorylation*) as the filter in each corpus, and we similarly restrict the generation of negative instances to those combinations that are associated at least once with an annotated semantic type combination in the set. For example, *methylated* →*histone H3* in Figure 6 is not included amongst the negative instances, since its semantic type combination, *Methylation*-*Protein*, never appears in the set of annotated semantic type combinations of GE. The filter applies similarly to other negative instances that would be erroneously created if a naïve corpus merge were used. For the hedge detector, all the instances are used without filtering, since all events are annotated with hedges. Figure 6 also shows that the positive examples are merged without filtering and that the number of shared positive instances, e.g., *lysine 4*:*Entity*, is increased. We note that this filtering approach is not perfect, and some correct negative examples are removed in Figure 6.

In training the EventMine models using multiple corpora with the filtering approach, we train separate one-versus-rest models for each type, as the set of negative examples that can be applied (that are not filtered) varies depending on the type under consideration, as mentioned previously. In addition to the outlined benefits of our approach in isolated classification settings, we note that in pipeline architectures such as EventMine, the use of merged sets of corpora is also expected to have cumulative benefits in terms of the ability to make use of additional features from instances predicted by preceding modules.

### Evaluation settings

#### Corpora

We employed the seven corpora introduced above. We used the standard train/development/test data splits provided for the GE, EPI, ID, and MLEE corpora. The DNAm and EPTM corpora only define train/evaluation splits, which were applied in all experiments on these corpora. As the mTOR corpus is not provided with a specific way to divide the data, we split the corpus into random train and evaluation sets on the document level.

We unified certain aspects of the corpus annotation that did not feature explicit direct overlap so that we can evaluate the results with the same criteria applied in the BioNLP ST 2011. The *Catalysis* event type in mTOR was replaced with the *Positive regulation* type, as the *Positive regulation* type in the other corpora is compatible with the combination of those event types in mTOR [7]. We also replaced *DNA domain or region* or *Protein domain or region* in MLEE with the genetic *Entity* type applied in the other resources [2]. We unified minor, semantically non-significant differences in role type names (e.g., *toLoc* →*ToLoc*, *Theme1* →*Theme*) among the corpora. We also filtered out rare role types (removing *fromLoc* and *product* from mTOR and MLEE) since they do not appear in all corpora and the arguments of the role types are not *Protein*.

Finally, we removed event structures appearing only once in the training data (e.g., *Binding* with eight *Theme*s in mTOR) to reduce computational cost.

#### Compared methods

We compare the following methods to training given multiple corpora: **Single**: consider each corpus separately, and learn separate models. **Merge**: merge the corpora with no modification, and learn a single model. **Stacking**: consider each corpus separately, using outputs of models trained on the other corpora as additional features, as done in e.g., [21], producing separate models for each corpus. **EasyAdapt**: apply the feature-based domain adaptation method of [23]. This method employs two kinds of features, corpus-dependent and corpus-independent features, and learns a single model from all the corpora. The set of features is duplicated for each instance, and one set is used as corpus-dependent and the other is used as corpus-independent features. Although this method produces a single model, the application of the model is corpus-dependent due to the corpus-dependent features. Here, we additionally treat the abstract and full-text portions of GE separately, following [20]. **Multiple**: learn a single model from all the corpora using the filtering approach proposed in this study. Table 4 summarises the differences among the methods.

**Table 4:** Characteristics of compared methods

**Characteristics**	**Single**	**Merge**	**Stacking**	**EasyAdapt**	**Multiple**
Learning from multiple corpora		x	x	x	x
Instance addition		x		x	x
Single corpus-independent model		x			x
Filtering falsely created instances					x

#### Event extraction settings

We follow the settings used by [21] for EventMine, with the following four exceptions. Firstly, we do not employ the protein-specific coreference resolver to avoid negative effects on named entities other than *Protein*. The extension of the coreference resolver to other named entity types is left as future work.

Secondly, we do not employ cross validation in training, to avoid negative effects that cross validation tends to ignore rare event types with small numbers of training instances.

Thirdly, we treat all post-translational modification (PTM) types as a single type in modules other than the trigger detector, which assigns the final type. This is a straightforward extension of the previously proposed EPI setting. Finally, in addition to several lexical and semantic resources, we use meta-knowledge cues from the GENIA meta-knowledge corpus [30,31] in the hedge detector. For brevity, we do not explain the details of the settings; we refer the reader instead to [21].

Evaluation on each corpus is performed after removing from the system output all the events involving event types not within the annotation scope of that corpus. We evaluate the results with the official evaluation tools and servers of the BioNLP ST 2011, and we mainly report results for the primary (“FULL”) evaluation criteria, which evaluate both whole event structures and their hedge types.

## Results and discussion

### Performance on annotated events

Tables 5 and 5 show the results of evaluation on the development sets of the various corpora. With regard to the overall performance on all the corpora (Table 5), the proposed approach, Multiple, achieves the best performance for both the event extraction and the hedge detection tasks. The performance for the Multiple setting is statistically significantly better than for the Single, Merge, and Stacking settings (p <0.001), using the approximate randomisation test [32]. The Multiple setting produced high recall results, which is arguably preferable for many applications, such as semantic search. In contrast, the F-Score for event extraction with the Merge setting is slightly lower than for the Single setting, and the overall F-Score with the Merge setting is effectively identical to that for the Single setting. This shows that the spuriously created negative examples cancel out the benefit of the increased training instances in the Merge setting. EasyAdapt appears to perform slightly worse than Multiple, although the difference is not statistically significant (p=0.28).

**Table 5:** Recall / precision / F-scores on the development portions of all the corpora

**Setting**	**Event**	**Hedge**	**Total**
Single	49.2/56.1/52.4	22.7/42.5/29.5	46.5/55.2/50.5
Merge	44.4/**63.6**/52.3	24.6/**50.4**/33.1	42.3/**62.6**/50.5
Stacking	50.2/56.7/53.3	24.3/42.2/30.9	47.5/55.7/51.3
EasyAdapt	50.7/58.4/54.3	25.4/48.7/33.4	48.1/57.8/52.5
Multiple	**51.2**/58.5/**54.6**	**31.5**/44.8/**37.0**	**49.2**/57.4/**53.0**

The FULL task evaluation criteria are employed. Event, hedge and their total results are shown. The highest results are shown in bold, and the lowest results are underlined.

**Table 6:** F-scores on the development portions of the corpora

**Setting**	**GE**	**ID**	**EPI**	**DNAm**	**EPTM**	**mTOR**	**MLEE**
Single	50.9	48.2	54.6	72.4	44.0	47.1	46.2
Merge	48.8	49.8	59.3	75.9	51.6	48.5	42.3
Stacking	51.3	50.3	56.6	72.4	44.6	48.6	47.1
EasyAdapt	51.3	**53.3**	58.4	75.6	49.7	45.3	47.6
Multiple	**52.7**	50.1	**59.6**	**76.0**	50.0	**51.0**	47.2

The FULL task evaluation criteria are employed. The highest results are shown in bold, and the lowest results are underlined.

In the detailed results separated by corpus (Table 6), we find that the performance on GE and MLEE using the naïvely merged corpora (Merge) shows a significant degradation compared to the single corpus results, although an improvement was observed for some of the other corpora. This indicates that the negative effect of simple corpus merging depends on the specific case considered. Our new method (Multiple) and Stacking are the only approaches that consistently improve performance over the Single setting. The stable improvement of Stacking is expected, as stacking trains a model on each target corpus and only adds new information compared to Single. The stable improvement of Multiple shows that the proposed filtering approach was effective in reducing the detrimental effects of spuriously created negative examples. Furthermore, the Multiple setting performed better than Stacking on 6 out of 7 corpora, which shows that direct instance addition in Multiple is usually better than the indirect use of information through stacking. EasyAdapt achieved good performance on ID and MLEE and showed comparable performance to Multiple on most of the corpora. In summary, the Multiple setting achieved the best results on GE, EPI, DNAm and mTOR, the EasyAdapt setting performed best on ID and MLEE, and the Merge setting performed best on EPTM. Taken together, the results in Tables 3 and 6 also show that the number of shared events does not necessarily correlate with the improvement achieved by the Multiple setting, even if the ratio of the number of increased events to the number of original events is quite large, such as for mTOR (Table 3). This effect is not unexpected, considering the various challenges of extracting each event type and the diverse distribution of the shared event types.

As shown in Figure 3, there are several event types that are isolated – that is, occur in only one corpus – as well as several event types that overlap between multiple corpora. To determine the effect of the various approaches to corpus combination for isolated vs. overlapping event types, we evaluated performance on these two sets of events separately. The results are summarised in Table 7. Here, we also show the results on isolated types excluding the PTM types, i.e., types in EPI and EPTM, since all PTM types share the same event structures and are thus not completely isolated. The results show that the Multiple setting not only improves the performance on the overlapping event types but also slightly improves the performance on the isolated event types. The approach is shown to be particularly effective for isolated non-PTM types, for which approaches other than Multiple resulted in decreased performance compared to Single. This shows that the instances introduced by the Multiple setting worked at least to some extent as useful negative instances for those isolated types.

**Table 7:** F-scores on isolated and overlapping types on the development portions of all the corpora

**Setting**	**Isolated (excluding PTM)**	**Isolated (all)**	**Overlap**
Single	59.1	55.5	51.8
Merge	57.4	55.7	51.5
Stacking	58.7	55.2	52.9
EasyAdapt	58.6	**56.1**	53.9
Multiple	**59.3**	**56.1**	**54.3**

The FULL task evaluation criteria are employed. Isolated types excluding PTM, all isolated types and overlapping types are shown. The highest results are shown in bold, and the lowest results are underlined.

Table 8 shows the performance on the held-out test portions of the four corpora that define a train/development/test split: GE, EPI, ID and MLEE. We note that the results on MLEE are not fully comparable with previously published results due to the modifications made to this corpus (see Corpora section). By contrast, the evaluation for GE, EPI and ID is identical to that applied in previous work on these resources. The test set results are in agreement with the evaluation on the development portion of the four corpora (Table 6) in that the settings with the highest results for each corpus are identical. The development and test evaluations also both indicate that Merge performs worse than Single on MLEE, and that the proposed approach, Multiple, performs better than Single and Merge on all four corpora. There are also some differences, for example in that Stacking and EasyAdapt give lower results than Single on GE, and Merge gives the lowest F-Score on ID. Despite such differences on the relative ranking of some methods for specific corpora, these results support the primary findings of evaluation on the development data that Multiple can consistently improve performance, and EasyAdapt or Multiple perform best on these four corpora.

**Table 8:** Recall / precision / F-scores on the test portions of the corpora

**Setting**	**GE**	**ID**	**EPI**	**MLEE**
Single	50.24/63.97/56.28	54.39/61.41/57.69	39.48/63.47/48.68	45.37/**61.20**/52.11
Merge	**51.31**/63.79/56.88	61.59/53.37/57.19	48.62/60.62/53.96	47.94/56.19/51.74
Stacking	49.67/64.72/56.21	54.60/61.89/58.02	40.13/66.19/49.97	**51.71**/54.77/53.20
EasyAdapt	49.72/63.19/55.65	58.96/**61.33**/**60.12**	44.70/**65.70**/53.21	51.11/55.73/**53.32**
Multiple	51.25/**64.92**/**57.28**	**64.01**/54.82/59.06	**54.28**/54.42/**54.35**	50.51/55.22/52.76

The FULL task evaluation criteria are employed. We report the subtask Task 1 (core argument detection) result for GE. The highest results are shown in bold, and the lowest results are underlined.

Although the performance differences between EasyAdapt and Multiple are not significant, there is a very important difference between the systems resulting from training using these two approaches. The Multiple setting produces a single system that operates on all the corpora, while the other most successful settings, i.e., Stacking and EasyAdapt, are corpus dependent (Table 4). Stacking produces multiple models, each specialised to a target corpus. EasyAdapt produces a single model, but the features for each instance depend on the target corpus. The consistent improvement demonstrated by our new approach is particularly notable considering that it results in a single, coherent system. The ability to produce such a system is important for practical applications, since it means that the simple application of one system can produce coherent analyses over a large part of the semantic space and avoids the need to combine system outputs for further processing.

We have shown here that the Multiple setting can produce a single, coherent system with highly competitive performance, but the approach still has some limitations. The approach is not as general as methods such as stacking, since it requires task-dependent filters. To further reduce the requirements to apply the method, we can consider ways to easily define filters appropriate for specific tasks, e.g., by providing templates. The setting can filter out correct negative examples, as shown in Figure 6, but we could further incorporate thesauri and dictionaries to extend the filtering dictionaries and relax the filtering. We have treated event types as mutually-exclusive, but event types in different corpora could also overlap or be hierarchically related. These cases would need to be treated separately. To further improve the approach, we thus need to consider ways to extend our method to be able to treat relations among types. For example, we could remove positive examples of overlapping types from negative examples, and merge more specific (child) positive instances to more general (parent) positive instances in a type hierarchy.

### Comparison with other event extraction systems

The results achieved on the three corpora of the BioNLP ST 2011 using our new method (Multiple) are summarised in Table 9, where the performance is compared to the two top performing systems in the shared task, i.e., FAUST [33] and the Turku Event Extraction System (TEES) [8], as well as to the version of EventMine incorporating coreference resolution and domain adaptation (EM-CR) [21]. FAUST combines two event extraction systems, a dual decomposition-based event extraction system [17] and a dependency parsing based event extraction system [18]. FAUST also merges GENIA and ID, using two copies of ID and one of GENIA for training a model for ID, corresponding to the Merge setting with the addition of different weights for instances from the two corpora. TEES is a pipeline-based event extraction system using SVMs with a similar overall architecture to EventMine. EM-CR performs better than the system with the Multiple setting on GE, since it employs the coreference resolution system to *Protein* entities, which was not employed in this paper (see the *Evaluation Settings* section of *Methods*). We also include the performance of EasyAdapt in the table as EasyAdapt achieved the best result on ID, as mentioned in the previous section.

**Table 9:** Recall / precision / F-scores on the test portions of BioNLP ST 2011 corpora

**System**	**GE**	**ID**	**EPI**
Multiple	51.25/**64.92**/57.28	**64.01**/54.82/59.06	**54.28**/54.42/**54.35**
EasyAdapt	49.72/63.19/55.65	58.96/61.33/**60.12**	44.70/**65.70**/53.21
EM-CR	**53.35**/63.48/**57.98**	60.55/54.97/57.63	49.06/55.39/52.03
FAUST	49.41/64.75/56.04	48.03/**65.97**/55.59	28.88/44.51/35.03
TEES	49.56/57.65/53.30	37.85/48.62/42.57	52.69/53.98/53.33

The official scores of the 2 top performing systems, FAUST and the Turku Event Extraction System (TEES), as well as EventMine with coreference resolution and domain adaptation (EM-CR), are shown for reference. We report the subtask Task 1 (core argument detection) result for GE. The highest scores are shown in bold.

We also note that other systems use three corpora at most, while our system uses seven corpora. Nevertheless, the results shown in Table 9 are encouraging, especially since our system with the Multiple setting is a single system, while other systems are separately tuned to each target corpus. The results further show that the single system built with the Multiple setting performs better than the highest published results on both of two established benchmark tasks ID and EPI.

### Analysis on additional event types

Finally, to evaluate performance on event types that can be extracted by the single system built with the Multiple setting, but are not annotated in the shared task corpora, we have manually evaluated 261 event instances extracted using the Multiple setting on the development portions of three corpora: 100 events for GE, 100 for EPI, and 61 for ID (Only 61 events of types not annotated in the ID corpus were predicted for the ID dataset). Table 10 shows the evaluation results. We find that the instances judged correct or acceptable give an estimated precision of 67.4% (176/261). This result is higher than for evaluation against the gold standard using FULL evaluation criteria. Whilst this result may in part reflect the looser matching criteria (i.e., “Acceptable”) applied during the manual evaluation, this high precision nevertheless lends further support to the broad applicability of the single system.

**Table 10:** Manual evaluation results on 261 event instances out of annotation scope

	**GE**	**ID**	**EPI**	**TOTAL**
Correct (Strict match)	65	23	63	151
Acceptable (Loose match)	8	1	16	25
Incorrect	27	37	21	85

## Conclusions

This paper has presented an approach to the construction of a wide coverage information extraction system through training on multiple corpora with partially overlapping annotation scopes. The approach heuristically detects and filters out unannotated parts from each corpus in an automated manner, so as to reduce the generation of spurious negative instances of types that are specific to particular corpora. The remaining instances are directly combined to make full use of the available annotated information. The approach was implemented in the EventMine system and evaluated on seven corpora, including three established BioNLP Shared Task (ST) resources. For all seven corpora, our approach improved the performance of the system sufficiently to achieve results better than those of systems trained on individual corpora. Our approach overcomes the problem of inconsistent annotation scopes in different corpora, and achieves comparable or higher performance than domain adaptation methods that produce separate models for each corpus. Training using the approach proposed in this paper produces a single system, and thus eliminates the need to combine results produced by multiple systems, each trained on a single corpus. Evaluation on BioNLP ST 2011 data showed that our system outperforms previously published systems on two out of three considered tasks. The wide-coverage event extraction system is made available as both a demo and a RESTful web service [34]. In future work, we will apply this system to the entire set of PubMed articles to enrich domain applications that make use of event extraction results, such as semantic search engines. This task is comparatively straightforward as the resulting system is not restricted to any individual corpus but can identify all targeted events in a single application. We will also extend and apply the method to other statistical NLP tasks such as named entity recognition.

## Competing interests

The authors declare that they have no competing interests.

## Authors’ contributions

All authors contributed to the production of the manuscript. SP, TO and SA supervised all steps of the work. MM built the system and carried out the experiments, and TO performed the manual evaluation. All authors read and approved the final manuscript.
